# The Trajectory and the Related Physical and Social Determinants of Body Mass Index in Elementary School Children: Results from the Child and Adolescent Behaviors in Long-Term Evolution Study

**DOI:** 10.1155/2014/728762

**Published:** 2014-07-08

**Authors:** Li-Ju Lin, Hsing-Yi Chang, Dih-Ling Luh, Baai-Shyun Hurng, Lee-Lan Yen

**Affiliations:** ^1^Department of Health, Taipei City Government, Taipei City 11008, Taiwan; ^2^Graduate Institute of Health Policy and Management, College of Public Health, National Taiwan University, Taipei 10055, Taiwan; ^3^Institute of Population Health Sciences, National Health Research Institutes, Miaoli County 35053, Taiwan; ^4^School of Public Health, Chung Shan Medical University, Taichung 40201, Taiwan; ^5^Department of Family and Community Medicine, Chung Shan Medical University Hospital, Taichung 40201, Taiwan; ^6^Surveillance and Research Division, Health Promotion Administration, Ministry of Health and Welfare, Taichung 40341, Taiwan

## Abstract

This study explored developmental trajectory patterns of BMI and associated factors. Participants included 1,609 students who were followed from age 7 to 12 years. Data collection involved annual self-administered questionnaires and records of height and weight. An ecological model was used to identify the factors associated with BMI trajectories. Group-based trajectory models and multinomial logit models were used in the statistical analysis. There were gender differences in BMI trajectories. Among boys, four BMI trajectories were normal or slightly underweight, persistently normal weight, overweight becoming obese, and persistently obese. Among girls, four BMI trajectories were persistently slightly underweight, persistently normal weight, persistently overweight, and persistently obese. The mean BMI in each trajectory group demonstrated an upward trend over time. In boys, BMI trajectories were significantly associated with after-school exercise, academic performance, family interactions, overweight parents, and father's education level. In girls, BMI trajectories were significantly associated with television viewing or computer use, family interactions, peer interactions, and overweight parents. Children under age 7 years who are already overweight or obese are an important target for interventions. The different factors associated with BMI trajectories can be used for targeting high risk groups.

## 1. Introduction

Childhood overweight and obesity is increasing worldwide [1, 2], and children who are overweight or obese face increased health risks such as hyperlipidemia, high cholesterol, high blood pressure, and type 2 diabetes [3–5]. Obesity has a long-term impact on health and research has shown that children who are overweight or obese are more likely to be obese as adults [6–8]. Therefore, childhood obesity is an important health problem that demands attention.

Currently, our understanding of developmental trajectories of childhood overweight is still limited. Longitudinal research examining trajectories of overweight and obesity in children is extremely important as childhood is the key developmental period during which risk factors for obesity arise. Moreover, a growing number of children are presenting with overweight or a rapid increase in body mass index (BMI) which if it continues into adolescence and adulthood will result in overweight [9]. Childhood is a key period for BMI development and children who have been overweight or obese are more likely to continue to be overweight or obese later in life. In contrast, children who maintain a BMI within the normal range are less likely to become overweight or obese later in life [10]. This indicates that there are likely to be distinct BMI trajectories within the population. Recent longitudinal research has categorized BMI developmental trajectories into groups based on the presence or absence of overweight or obesity [11–13]. Another study has used BMI to categorize BMI trajectories in girls [14]. However, despite this past research demonstrating that children can be grouped into distinct BMI trajectory types, there has been little research investigating potential sex differences in BMI developmental trajectories.

It is essential to consider the influence of an obesogenic environment when considering factors associated with the development of childhood overweight and obesity [15]. Past research supports the use of an ecological model to analyze the multiple factors associated with obesity [16, 17]. An ecological model [18] is a comprehensive and broad conceptual approach that emphasizes the multiple levels of factors that can influence health behavior. However, in the majority of past longitudinal research on factors associated with BMI developmental trajectories in children, few studies have considered multiple levels of factors such as modifiable diet and exercise in school-age, the school level, or community level. As a result, to date, there has been only a limited examination of factors associated with BMI trajectories. Moreover, the change in these associated factors during follow up and the impact of this change on BMI trajectories have been rarely considered. As a result, we have adopted an ecological model in the present study to examine multiple levels of factors associated with BMI trajectories. Potential factors were chosen from the different levels of individual, family, school, and community. In addition, longitudinal change in these variables was incorporated into our investigation of factors associated with BMI developmental trajectories in children.

Therefore, the main aims of our study were (1) to describe BMI developmental trajectories in our sample from grade 1 to grade 6 and to determine the presence of any sex differences; and (2) to determine factors associated with BMI developmental trajectories in children at the individual, family, school, and community level.

## 2. Methods and Procedures

### 2.1. Study Sample

Our study analyzes data from the child and adolescent behaviors in long-term evolution (CABLE) project, a cohort study that commenced in 2001 [19]. The baseline CABLE sample comprised of first grade students from 18 randomly selected schools. Following the baseline survey in 2001 when the school children were in grade one, children and their parents who agreed to participate were followed yearly with a child and parent questionnaire. Data was also collected from the annual school health check conducted on the children. Data were collected using questionnaires specifically designed for the CABLE project, which have been assessed previously for reliability and validity [19]. Questionnaires were self-completed by the children and their parents.

The baseline CABLE sample comprised 2215 children. The present study includes those participants who were followed from grade one (2001) to grade six (2006) and had complete data for height and weight. We also excluded participants with missing data for father's and mother's height and weight, resulting in a final sample of 1609 children for our analysis (50.34% boys and 49.66% girls). The proportion of boys and girls in our sample was not significantly different from that at baseline (51.20% boys and 48.40% girls).

### 2.2. Study Variables

The dependent variable in our analysis was the BMI developmental trajectory groups. BMI (weight/height^2^) was calculated using height and weight data collected as part of the annual school health check from 2001 to 2006. These annual estimations of BMI were then used to examine BMI trajectories.

Independent variables were taken from the child questionnaires (data from 2001 to 2006 were taken) and school survey. Multilevel variables included (1) the individual factors including residential location, self-perceived academic performance (data from 2003 to 2006 were taken), and time-varying behavioral factors including eating breakfast, eating fruit and vegetables, eating fast food, drinking sugary drinks, participating in after-school exercise, television viewing or computer use patterns, and staying up late. We measured the frequency of these behaviors in the past week (data from 2001 to 2006 were analyzed); (2) family factors included family interactions (data from 2001 to 2006 was taken), parent's behavioral supervision (data from 2003 to 2006 was taken), and peer interactions (data from 2005 to 2006 was taken); (3) school factors included the presence of a consumer's cooperative and recreational facilities; and (4) community factors included perceived neighborhood interactions and perceived neighborhood safety (data from 2005 to 2006 was taken). Control variables included level of pubertal development, parent's overweight, household monthly income, father's education level, and mother's education level. Parent's overweight was estimated by using the self-reported height and weight from the parent's questionnaire to calculate BMI. The presence of overweight was then determined based on national standardized BMI cut-points. The reliability and validity of the scales for family interactions were assessed. Reliability was measured by internal consistency and validity was assessed by exploratory factor analysis.

Based on our study aim of examining trajectories in BMI in our sample over 6 years, we incorporated the 6 annual measurements of BMI into a group-based trajectory model. Group-based trajectory model is a semiparametric model by Nagin et al. [20–22]. This model aims to classify individuals into groups according to their patterns over time. This method is different from classifying individuals into groups based on their baseline values, which did not account for the longitudinal pattern.

The statistical model is described as follows:

(1) P(Yi)=∑j∏jPj(Yi).

*P*(*Y*_*i*_) denotes the probability of *Y*_*i*_. *Y*_*i*_ represents the longitudinal sequence of measurements on an individual *i* over *t* periods. *P* ^*j*^(*Y*_*i*_) is the probability of *Y*_*i*_ given membership in group *j*. ∏_*j*_  is the probability of group *j*. Consider

(2) $$\begin{array}{c} Y_{it}^{j}=\beta_{0}^{j}+\beta_{1}^{j}{\text{age}}_{it}+\beta_{2}^{j}{\text{age}}_{it}^{2}+\beta_{3}^{j}{\text{age}}_{it}^{3}+\varepsilon . \end{array}$$

*Y*_*it*_^*j*^ is a latent variable for individual *i*'s age at time *t* given membership in group *j*. *β*_1_, *β*_2_, and *β*_3_ are the parameters of the pattern of trajectory. *ε* is a disturbance assumed to be normally distributed with zero mean and constant variance *σ* ^2^.

It assumes that distinct trajectory groups exist as opposed to a heterogeneous individual distribution of long-term trajectories in the population. During analysis, the sample was divided into a number of groups and distinct groups of BMI developmental trajectories were selected based on model fit indices. The most appropriate model was selected based on the highest BIC value (2ΔBIC > 10), a posterior probability of greater than 0.70 and a trajectory group size of at least 5% of the sample.

In this study a number of factors associated with BMI trajectories change over time. These factors include eating breakfast, eating fruit and vegetables, drinking sugary drinks, eating fast food, participating in after-school exercise, television viewing and computer use patterns, staying up late, self-perceived academic performance, family interactions, parent's behavioral supervision, and peer interactions. Therefore, we included data on changes in these variables from grade one to six and divided them into different groups when comparing different trajectory groups.

A multinomial logit model was used to examine factors associated with BMI developmental trajectories in the sample. The dependent variable was BMI trajectory groups. We examined differences in factors associated with particular BMI trajectory groups compared to the reference trajectory group among boys and girls, respectively.

## 3. Results

Baseline demographic characteristics of the study sample are shown in Table 1. There were 810 boys (50.34%) and 799 girls (49.66%) in the sample. With regard to parent's overweight, having an overweight father was the most common (43.69%). The most common monthly household income was high income at 48.42%. The most common level of education was college and above in both fathers (59.85%) and mothers (47.91%).

### 3.1. Developmental Trajectories of BMI in the Study Sample

BMI values over the six years of follow up were used to create the BMI developmental trajectories. Trajectories were selected based on the largest BIC value, 2ΔBIC greater than 10, a posterior probability of greater than 0.70, and a trajectory group size of at least 5% of the study sample. This resulted in four distinct BMI trajectory groups. The mean BMI for each age group and the mean BMI of each trajectory group increased over time. The chi-squared test demonstrated a statistically significant sex difference in the distribution of BMI trajectory groups (*χ* ^2^ = 30.23, *P* < 0.001), and as a result, we stratified our analyses by sex.

**Figure 1:**
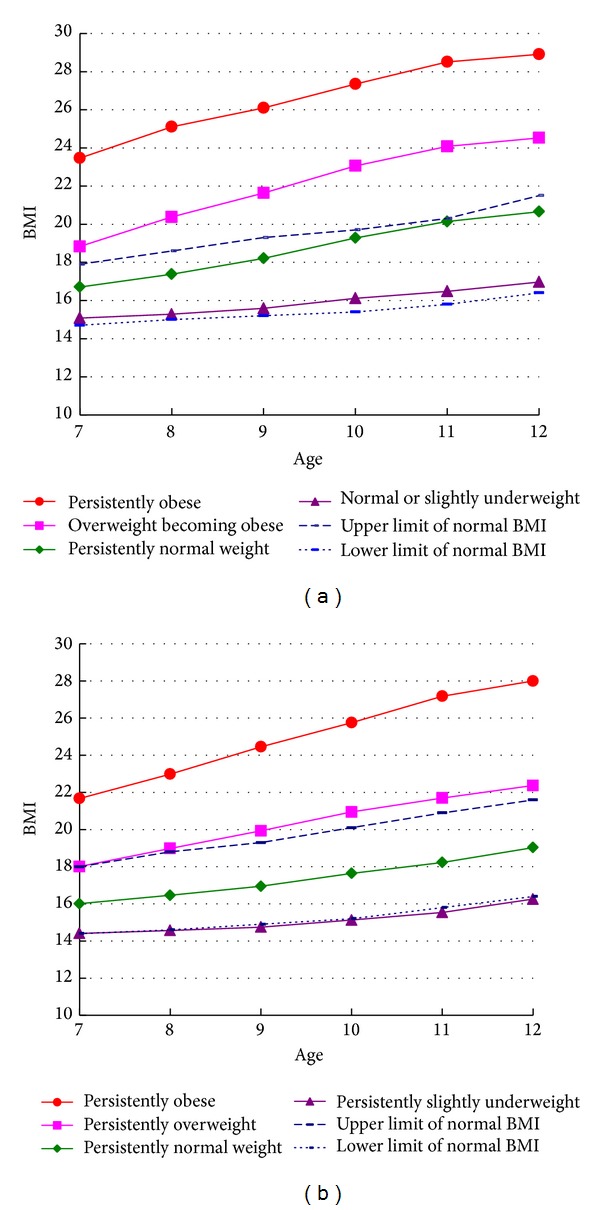
BMI developmental trajectory groups in boys and girls from ages 7 to 12 years (upper graph is boys and the lower graph is girls).

We used group-based trajectory modeling to analyze the sample, using the abovementioned criteria for model selection. BMI developmental trajectories were separated by sex and the mean BMI for each trajectory group was compared with national standard cut-points for overweight and obesity. The trajectory groups were then named accordingly.  Table 2 shows the distribution of mean BMI by BMI trajectory group and age group in boys. There were four BMI trajectory groups in boys: normal or slightly underweight (40.62%), persistently normal weight (34.69%), overweight becoming obese (18.15%), and persistently obese (6.54%) (Figure 1). The mean BMI of each trajectory group increased with age.  Table 3 shows the distribution of mean BMI by BMI trajectory group and age group in girls. There were also four BMI trajectory groups in girls: persistently slightly underweight (31.04%), persistently normal weight (40.18%), persistently overweight (22.03%), and persistently obese (6.76%). Figure 1 shows the BMI developmental trajectories in boys and girls and demonstrates that the mean BMI in each trajectory group increased with age.

Multinomial logit modeling was used to examine the relationship between individual, family, school, community, behavioral factors, and BMI developmental trajectories in the study sample. The persistently normal weight trajectory group was taken as the reference group, against which the other trajectory groups were compared. The various multilevel, behavioral, and control variables were entered into the model and those that remained statistically significant were retained in the final multinomial logit model. The results are stated below.

### 3.2. Factors Associated with a Persistently Obese or Overweight Becoming Obese BMI Trajectory in Boys

We found that boys with an overweight father (OR = 4.23), an overweight mother (OR = 4.68), or with both parents overweight (OR = 8.22) were more likely to follow a persistently obese trajectory than a persistently normal weight trajectory. Boys who had a low level of participation in after-school exercise were also more likely to have a persistently obese trajectory (OR = 3.76) compared to boys who frequently participated in after-school exercise. Boys with a low perceived academic performance were 2.30 times as likely to have a persistently obese BMI trajectory compared to boys with high perceived academic performance.

We found that boys with fathers with a college or higher level of education were less likely (OR = 0.44) than boys with fathers with a junior high school or below level of education to follow an overweight becoming obese BMI trajectory than a persistently normal weight trajectory. In addition, boys with persistently high levels of family interactions were 2.32 times as likely to have an overweight becoming obese BMI trajectory compared to boys with persistently low family interactions (Table 4).

### 3.3. Factors Associated with a Persistently Obese and a Persistently Overweight BMI Trajectory in Girls

We found that girls whose parents were both overweight were more likely to have a persistently obese BMI trajectory (OR = 6.84). Girls who frequently watched television or used the computer were also more likely to have a persistently obese trajectory (OR = 4.03) compared to those with low levels of television viewing or computer use. In addition, girls whose frequency of peer interactions changed from high to low levels were more likely to have a persistently obese BMI trajectory (OR = 2.22) compared to those with persistently high levels of peer interactions.

We found that girls whose fathers were overweight (OR = 1.75) or for whom both parents were overweight (OR = 2.31) were more likely to have a persistently overweight BMI trajectory. Girls who frequently watched television or used the computer were also more likely to have a persistently overweight BMI trajectory (OR = 2.26) compared to girls with low levels of television viewing or computer use. In addition, girls who maintained a medium level of family interactions were more likely to have a persistently overweight trajectory pattern than girls who maintained a low level of family interactions (OR = 1.72) (Table 4).

## 4. Discussion

In this study we used a longitudinal data to classify children by their long-term BMI status and examined associated factors in school children. We found that there were four types of BMI trajectory and the mean BMI in each group increased over time. Our key finding was that BMI status persisted from grade 1 to 6. In addition, BMI trajectory types differed between boys and girls. Boys had a high possibility going from overweight to obesity than girls. Using an ecological model, we included a wide range of variables over multiple levels. There were differences in the factors associated with the patterns between boys and girls. In boys “overweight becoming obese” was associated with higher family interactions and lower father's education level, whereas “persistently obese” was associated with lower frequency after-school exercise, lower self-perceived academic performance, and parent's overweight. In girls, persistently higher television viewing or computer use and parent's overweight were associated with an overweight or obesity trajectories. In addition, “persistently overweight” was associated with persistent medium family interactions and “persistently obese” was associated with unstable peer interactions.

In this study we found that the mean BMI of school children increased over time from grade one to grade six in both boys and girls. Similar to the present study, a previous study has also found distinct BMI trajectory groups in girls [14]. However in this previous study, one of the trajectory patterns observed involved an increase in mean BMI followed by a later decrease at about the age of 9 years. In contrast, we found that the mean BMI increased with age across all trajectory groups. It is possible that these differences could be related to the earlier onset of puberty in western countries, with associated effects on BMI developmental trajectories. Both the mean BMI and the BMI growth curve demonstrated an increasing trend, emphasizing the importance of paying close attention to children's BMI during this period. However, it is also important to note that BMI trajectory patterns can change during puberty, and, therefore, these findings should be confirmed further by future research.

In contrast to previous research, the use of longitudinal BMI data in our study enabled us to demonstrate distinct BMI developmental trajectory groups in boys and girls. Past research has used BMI values to directly divide the study sample into overweight or obese categories and then examined patterns of developmental trajectories. These past researches did not found any gender differences in developmental trajectories [11] or had been conducted on a single gender group [14]. However, there was one group of boys going from overweight to obese, whereas the overweight girls persisted in overweight. The possible reason was that girls were more likely to pay attention to body image; then they made efforts to maintain their body weight [23].

We also incorporated a wide range of multilevel variables associated with BMI trajectories in the present study. The majority of past research investigating factors associated with BMI trajectories has focused on early life factors such as birth weight and mother's weight at delivery. In addition, the majority of studies have used baseline values or mean values when examining relationships with BMI trajectories. In contrast, the present study included individual behaviors as well as family, school, and community factors; we also considered changes over time in these variables in the multilevel model, which investigated factors associated with BMI trajectory groups. We found that boys who had persistently low levels of participation after-school exercise were more likely to have a persistently obese trajectory. One past study reported no relationship between an overweight trajectory and after-school exercise. However, they found that those who were less physically active were more likely to be in a everoverweight group (versus never overweight) [13]. We found a protective effect of greater after-school exercise. Boys who had lower satisfaction with their self-perceived academic performance were more likely to have a persistent obese trajectory compared to those who had higher satisfaction. A previous cross-sectional study has also found an association between self-reported low academic grades in students and high BMI, overweight, and obesity [24]. In addition, longitudinal research has reported a relationship between becoming obese and a decline in academic performance in children [25]. Future research could investigate the mechanisms behind this association between self-perceived academic performance and a persistently obese BMI trajectory and whether it is based on differences in physical activity or psychological stress.

Girls who had a high frequency of television viewing or computer use were more likely to have a persistently overweight or persistently obese BMI trajectory patterns. O'Brien et al. also found that a greater frequency of television viewing was associated with a greater likelihood of being in the elementary overweight group or in a constantly overweight group [13]. Research has also found that in children with overweight parents, a greater length of time spent by parents watching television or using the computer is associated with an increased likelihood of children being overweight. However, no significant association was observed when parents were not overweight [26]. In the present study, we found that after controlling for parent's overweight, television viewing or computer use remained significantly associated with BMI trajectories, confirming the negative impact of frequent television viewing and computer use on BMI trajectory. Future research could investigate the possible mechanisms behind this association such as the impact of a motionless state without physical activity or the influence of media advertising [27].

We found that girls whose frequency of peer interactions changed from high to low levels were more likely to have a persistently obese BMI trajectory compared to girls with persistently high peer interactions. Cross-sectional research has found that overweight children are more likely to experience psychological stress or be worried about their weight. Associations have also been observed between BMI and being made fun of by peers and girls report experiencing more psychological stress than boys [28]. It is possible that the decrease in peer interactions and the associated poor interpersonal relations or psychological stress leads girls to consume food as a way of relieving their emotions. Alternatively, these girls may be being made fun of by their peers because of their increased weight and for similar reasons have decided to participate less in physical activity or outdoor activities. Future research could establish whether a reduction in peer interactions leads to a persistently obese BMI trajectory in girls and the possible mechanisms behind this association.

We found that boys with persistently high family interactions were more likely to have an overweight becoming obese BMI trajectory compared to boys with persistently low family interactions. In girls, those with a persistently medium level of family interactions were more likely to have a persistently overweight trajectory compared to girls with persistently low family interactions. Zabinski et. al found that household dietary rules and family support influence the intake of dietary fat and fruit and vegetables by children [29]. Food intake by overweight or obese children is significantly associated with food intake by their parents [30]. Therefore, it is possible that a high level of certain family interactions has a negative impact on dietary behavior in children. Further research is needed to determine if the high frequency of family interactions indicates that meals are used to celebrate or reward particular events or that the family is eating together more frequently, resulting in a greater intake of food.

We found that parent's overweight was associated with BMI trajectories in their children. Moreover, we found that the greatest risk of a persistently obese trajectory occurred when both parents were overweight. The influence of parent's overweight on their child's BMI could be due to a combination of genetic and environmental factors. Overweight in very young children may be more related to genetic factors, whereas environmental factors may be more important for overweight following puberty [31]. We also found that boys whose fathers had a college or higher level of education were less likely to become overweight or obese compared to boys whose fathers had a junior high school or lower level of education. These findings are similar to those reported by Ventura et al. (2009) [14].

This study has several limitations. The presence of overweight or obesity in parents was based on the calculation of BMI from self-reported height and weight. As there was no direct measurement of parents' actual height or weight this may have led to some inaccuracies. In addition, as there were only two years of data available for the community variables of self-perceived neighborhood safety and neighborhood interactions, we were unable to look at the change in these variables over six years. We also did not have any data on social environmental factors such as the density of fast food outlets or the amount of green space in the local area, so we were unable to include these variables in our analysis.

In conclusion, we found that there were distinct BMI trajectory groups and associated factors from grade 1 to 6. We suggested attention should be given to the time before grade 1 for prevention of overweight and obesity. In addition to parent's overweight and father's education level, other factors such as individual behaviors, family, and school factors also play an important role. Boys with overweight parents, fathers with a lower level of education, and lower self-perceived academic performance are in need of particular attention. Girls with overweight parents and unstable peer interaction are in need of particular attention. The different factors associated with BMI trajectories in boys and girls can be used for targeting high risk groups. We suggested the interventions for boys should promote persistence higher exercise time after class, whereas, for girls, it is to reduce the amount of time spent on watching television or using the computer. The focus on food as a part of family and peer gatherings should be reduced with more emphasis on activities involving movement so that children develop good habits at an early age.

## Figures and Tables

**Table 1:** Distribution of demographic characteristics in the study sample.

Characteristic	Boys (*n* = 810)	Girls (*n* = 799)	Total (*n* = 1609)
	*n* (%)	*n* (%)	*n* (%)
Parent's overweight			
Neither	232 (28.64)	239 (29.91)	471 (29.27)
Father	350 (43.21)	353 (44.18)	703 (43.69)
Mother	75 (9.26)	64 (8.01)	139 (8.64)
Both	153 (18.89)	143 (17.90)	296 (18.40)
Household monthly income			
Low	123 (15.19)	120 (15.02)	243 (15.10)
Medium	286 (35.31)	301 (37.67)	587 (36.48)
High	401 (49.51)	378 (47.31)	799 (48.42)
Father's education level			
Junior high school and below	63 (7.78)	60 (7.51)	123 (7.64)
Senior high school	256 (31.60)	267 (33.42)	532 (32.50)
College and above	491 (60.62)	472 (59.07)	963 (59.85)
Mother's education level			
Junior high school and below	60 (7.41)	63 (7.88)	123 (7.64)
Senior high school	362 (44.69)	353 (44.18)	715 (44.44)
College and above	388 (47.90)	383 (47.93)	771 (47.91)

**Table 2:** Mean BMI by BMI trajectory group and age in boys.

Trajectory group	Group 1		Group 2		Group 3		Group 4	
	(normal or slightly underweight)		(persistently normal weight)		(overweight becoming obese)		(persistently obese)	
	*n* = 329 (40.62%)		*n* = 281 (34.69%)		*n* = 147 (18.15%)		*n* = 53 (6.54%)	
Age (years)	Mean	(95% CI)	Mean	(95% CI)	Mean	(95% CI)	Mean	(95% CI)
7.0	15.08	(14.89–15.19)	16.71	(16.51–16.89)	18.83	(18.51–19.01)	23.47	(22.93–23.66)
8.0	15.28	(15.22–15.43)	17.38	(17.20–17.53)	20.38	(20.22–20.60)	25.11	(24.71–25.20)
9.0	15.59	(15.54–15.77)	18.21	(18.09–18.44)	21.64	(21.61–22.02)	26.10	(26.08–26.59)
10.0	16.12	(15.93–16.17)	19.28	(19.05–19.42)	23.06	(22.79–23.20)	27.35	(27.23–27.76)
11.0	16.48	(16.36–16.61)	20.14	(19.90–20.30)	24.08	(23.73–24.13)	28.51	(28.12–28.65)
12.0	16.97	(16.80–17.15)	20.66	(20.46–20.94)	24.53	(24.37–24.89)	28.91	(28.62–29.40)

**Table 3:** Mean BMI by BMI trajectory group and age in girls.

Trajectory group	Group 1		Group 2		Group 3		Group 4	
	(persistently slightly underweight)		(persistently normal weight)		(persistently overweight)		(persistently obese)	
	*n* = 248 (31.04%)		*n* = 321 (40.18%)		*n* = 176 (22.03%)		*n* = 54 (6.76%)	
Age (years)	Mean	(95% CI)	Mean	(95% CI)	Mean	(95% CI)	Mean	(95% CI)
7.0	14.41	(14.26–14.57)	16.01	(15.83–16.13)	18.01	(17.94–18.28)	21.68	(21.36–22.08)
8.0	14.57	(14.41–14.63)	16.46	(16.34–16.58)	18.99	(18.85–19.14)	22.99	(22.73–23.25)
9.0	14.75	(14.64–14.88)	16.95	(16.86–17.13)	19.93	(19.75–20.01)	24.46	(24.24–24.71)
10.0	15.13	(15.00–15.24)	17.64	(17.46–17.75)	20.95	(20.63–20.90)	25.76	(25.68–26.16)
11.0	15.54	(15.49–15.74)	18.23	(18.13–18.42)	21.70	(21.49–21.81)	27.18	(26.86–27.38)
12.0	16.26	(16.07–16.41)	19.03	(18.82–19.18)	22.37	(22.34–22.73)	28.00	(27.48–28.21)

**Table 4:** Factors associated with BMI development trajectory groups in boys and girls.

	Boys^1^			Girls^2^	
Variable	Overweight becoming obese	Persistently obese	Variable	Persistently overweight	Persistently obese
	OR	OR		OR	OR
After school exercise			Television viewing or computer use		
Low becoming high/persistently high	1.08	2.47	Persistently medium/persistently low level	1.41	1.96
Persistently low/persistently high	1.14	3.76∗∗	Persistently high level/persistently low level	2.26∗	4.03∗
Self-perceived academic performance			Family interactions		
Low satisfaction/high satisfaction	1.36	2.30∗	Persistently medium level/persistently low level	1.72∗	1.21
			Persistently high level/persistently low level	1.39	1.06
Family interactions			Peer interactions		
Low becoming medium/persistently low	1.41	2.14	Medium becoming high/persistently high	1.02	1.49
Medium becoming low/persistently low	1.52	0.66	High becoming low/persistently high	1.23	2.22∗
Persistently high/persistently low	2.32∗	2.02			
Parent's overweight			Parent's overweight		
Father overweight/neither parent overweight	1.71	4.23∗	Father overweight/neither parent overweight	1.75∗	1.42
Mother overweight/neither parent overweight	2.56	4.68∗	Mother overweight/neither parent overweight	1.55	1.11
Both parents overweight/neither parent overweight	1.53	8.22∗∗	Both parents overweight/neither parent overweight	2.31∗∗	6.84∗∗∗
Father's education level					
Senior high school/junior high school and below	0.68	0.70			
College and above/junior high school and below	0.44∗	0.50			

^1^The OR in boys has been adjusted for after-school exercise, self-perceived academic performance, family interactions, peer interactions, parent's overweight, and father's education level. Reference group: persistently normal weight.

^2^The OR in girls has been adjusted for television viewing or computer use, family interactions, peer interactions, and parent's overweight. Reference group: persistently normal weight.

*P < 0.05; **P < 0.01; ***P < 0.001.
